# Supplementary material for: Aberrant DNA and RNA Methylation Occur in Spinal Cord and Skeletal Muscle of Human SOD1 Mouse Models of ALS and in Human ALS: Targeting DNA Methylation Is Therapeutic
Source: Cells. 2022 Oct 31;11(21):3448. doi: 10.3390/cells11213448 (PMC9657572; doi:10.3390/cells11213448)
Supplement: Supplementary file 1 [file cells-11-03448-s001.zip › cells-1977660-supplementary.pdf]

Table S1. Hypermethylated Gene Promoters in Skeletal Muscle of hSOD1-G37R^{mus} Transgenic Mice

Gene	Promoter	Protein Name
Pi15	LCP	peptidase inhibitor 15
Tcfap2b	ICP	transcription factor AP-2-beta isoform 1
Tcfap2b	ICP	transcription factor AP-2-beta isoform 2
Kcnq5	ICP	potassium voltage-gated channel subfamily KQT
Arhgef4	ICP	rho guanine nucleotide exchange factor 4
Mgat4a	LCP	alpha-1,3-mannosyl-glycoprotein
Aox4	ICP	aldehyde oxidase 4
Orc2	LCP	origin recognition complex subunit 2 isoform B
Creb1	HCP	cyclic AMP-responsive element-binding protein 1
Speg	ICP	striated muscle-specific
Trip12	HCP	thyroid hormone receptor interactor 12
Alppl2	LCP	embryonic-type alkaline phosphatase precursor
Ecel1	ICP	endothelin-converting enzyme-like 1
Fam132b	HCP	hypothetical protein LOC227358 precursor
Olfr1411	LCP	olfactory receptor 1411
Pdcd1	LCP	programmed cell death protein 1 precursor
Ptpn4	HCP	tyrosine-protein phosphatase non-receptor type
Faim3	LCP	fas apoptotic inhibitory molecule 3 precursor
Tnni1	LCP	troponin I, slow skeletal muscle
Tnni1	LCP	troponin I, slow skeletal muscle
Fmo2	LCP	dimethylaniline monooxygenase [N-oxide-forming]
Pvrl4	LCP	poliovirus receptor-related protein 4 isoform b
Tagln2	ICP	transgelin-2
Vsig8	LCP	V-set immunoglobulin domain contaning 8
Sccpdh	HCP	probable saccharopine dehydrogenase
AA408296	HCP	digestive organ expansion factor homolog
Syne1	LCP	nesprin-1 isoform 1
Iyd	LCP	iodotyrosine dehalogenase 1 precursor
Raet1d	LCP	retinoic acid early-inducible protein 1-delta
Taar2	LCP	trace amine-associated receptor 2
Taar4	LCP	trace amine-associated receptor 4
Taar5	LCP	trace amine-associated receptor 5
Smpd2	HCP	sphingomyelin phosphodiesterase 2
Bves	HCP	blood vessel epicardial substance
Edar	ICP	tumor necrosis factor receptor superfamily
Ccar1	HCP	cell division cycle and apoptosis regulator
Rhobtb1	ICP	rho-related BTB domain-containing protein 1
Ccdc6	HCP	coiled-coil domain-containing protein 6
Col18a1	ICP	collagen alpha-1(XVIII) chain isoform 2
Lrrc3	ICP	leucine-rich repeat-containing protein 3
Mier2	HCP	mesoderm induction early response protein 2
Odf3l2	LCP	outer dense fiber protein 3-like protein 2
Kiss1r	ICP	kiSS-1 receptor
Sbno2	HCP	protein strawberry notch homolog 2
Sbno2	HCP	protein strawberry notch homolog 2
Adamtsl5	ICP	ADAMTS-like protein 5 isoform 1
Adamtsl5	ICP	ADAMTS-like protein 5 isoform 2
Mex3d	HCP	RNA-binding protein MEX3D
Csnk1g2	LCP	casein kinase I isoform gamma-2 isoform 2

Diras1	ICP	GTP-binding protein Di-Ras1
Ncln	HCP	nicalin precursor
Sirt6	HCP	NAD-dependent deacetylase sirtuin-6 isoform 2
Ankrd24	LCP	ankyrin repeat domain-containing protein 24
Timp3	ICP	metalloproteinase inhibitor 3 precursor
Gli1	ICP	zinc finger protein GLI1
Nxph4	HCP	neurexophilin 4
Nab2	HCP	NGFI-A-binding protein 2 isoform 2
Myo1a	LCP	myosin-Ia
Rdh1	LCP	retinol dehydrogenase 1 (all trans)
Sdr9c7	LCP	short-chain dehydrogenase/reductase family 9C
Dnajc14	HCP	dnaJ homolog subfamily C member 14
1110012D08Ril	ICP	hypothetical protein LOC73827
Eif4enif1	HCP	eukaryotic translation initiation factor 4E
Eif4enif1	HCP	eukaryotic translation initiation factor 4E
Eif4enif1	HCP	eukaryotic translation initiation factor 4E
Smtn	ICP	smoothelin
Zmiz2	LCP	zinc finger MIZ domain-containing protein 2
Tns3	HCP	tensin-3
Vps54	HCP	vacuolar protein sorting-associated protein 54
C1qtnf2	ICP	complement C1q tumor necrosis factor-related
Irgm1	ICP	immunity-related GTPase family M protein 1
Olfr1387	LCP	olfactory receptor 1387
Scgb3a1	LCP	secretoglobin family 3A member 1 type B
Agxt2l2	HCP	alanine--glyoxylate aminotransferase 2-like 2
Gjc2	LCP	gap junction gamma-2 protein
Gjc2	LCP	gap junction gamma-2 protein
Jmjd4	HCP	jmjC domain-containing protein 4
Tom1l2	HCP	TOM1-like protein 2 isoform c
Lrrc48	HCP	leucine-rich repeat-containing protein 48
Aldh3a1	LCP	aldehyde dehydrogenase, dimeric NADP-preferring
Myocd	ICP	myocardin isoform B
Vamp2	HCP	vesicle-associated membrane protein 2
Per1	ICP	period circadian protein homolog 1
Per1	ICP	period circadian protein homolog 1
Hes7	LCP	transcription factor HES-7
Cldn7	LCP	claudin-7
Cldn7	ICP	claudin-7
Alox15	ICP	arachidonate 12-lipoxygenase, leukocyte-type
Chrne	LCP	acetylcholine receptor subunit epsilon
Gsg2	HCP	serine/threonine-protein kinase haspin
Olfr412	LCP	olfactory receptor 412
Coro6	LCP	coronin-6 isoform C
Rhot1	HCP	mitochondrial Rho GTPase 1 isoform 1
Slfn4	ICP	schlafen 4
Acaca	LCP	acetyl-CoA carboxylase 1
Ppm1d	HCP	protein phosphatase 1D
Dhx40	HCP	probable ATP-dependent RNA helicase DHX40
Cuedc1	HCP	CUE domain-containing protein 1 isoform 1

Figure S1

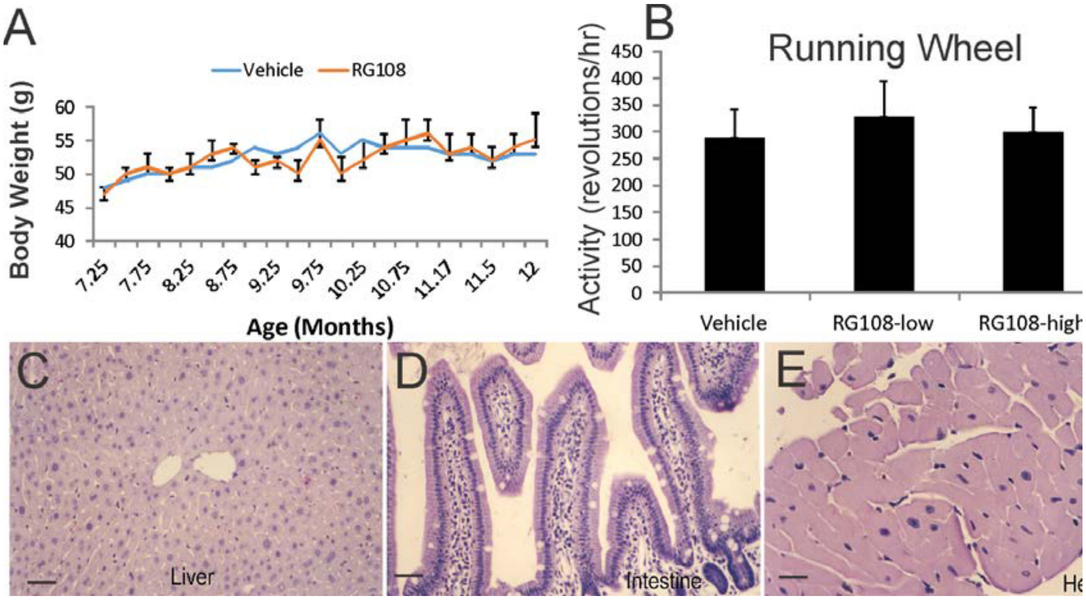

Figure S1. RG108 does not have overt off-target effects in vivo. **(A)** Non-tg mice were treated (2 mg/kg, ip) chronically with RG108 and were assessed for body weight. **(B)** Non-tg mice were treated (ip) chronically with low dose (2 mg/kg) and high dose (10 mg/kg) RG108 and were assessed for running wheel motor activity. (C-E) With high dose treatment, the liver (C), intestine (D), and heart (E) were histologically normal. Scale bars: 100 µm (A,B), 50 µm (C).
